# Vitamin D metabolism in critically ill patients with acute kidney injury: not a sole player

**DOI:** 10.1186/s13054-024-04965-5

**Published:** 2024-05-24

**Authors:** Alexandre Braga Libório

**Affiliations:** Medical Sciences Postgraduate Program, Universidade de Fortaleza-UNIFOR, Fortaleza, Ceará Brazil

**To the Editor,**

We commend Dr. Cameron et al. [1] for their insightful study on vitamin D metabolism in critically ill patients with acute kidney injury (AKI). The authors describe how the trajectory of 1,25-dihydroxyvitamin D generally mirrors renal function, decreasing its serum levels with the onset of AKI and increasing with AKI recovery. This pattern is distinct from that of 25-hydroxyvitamin D.

Although these results are strongly biologically plausible [2], given that the proximal tubular cells of the kidney convert 25(OH)D to 1,25(OH)_2_D, we need to consider the complexity of vitamin D metabolism before attributing the reduction in 1,25(OH)_2_D in AKI solely to tubular cell damage. Several mechanisms modulate the conversion of 25(OH)D to 1,25(OH)_2_D via 1-alpha-hydroxylase, with fibroblast growth factor-23 (FGF23) playing a crucial role in this process [3].

As the authors noted, a limitation of the study is the absence of FGF23 measurements. In a previous study, our group evaluated 25(OH)D, 1,25(OH)_2_D, serum phosphorus and FGF23 in a critically ill population [4], the great majority of whom had an estimated glomerular filtration rate above 45 mL/min/m^2^, like the above cited study. Before the clinical detection of AKI, FGF-23 levels were already elevated in patients who later developed AKI in comparison with those who did not develop AKI. Moreover, we found an inverse relationship between FGF23 and 1,25(OH)_2_D before AKI onset. Regarding phosphorus, another important regulator of 1,25(OH)_2_D production not evaluated by Cameron et al., we found a significant positive correlation between FGF-23 and serum phosphorus. These findings allow us to speculate that the increase in FGF-23 in critically ill patients occurs before the clinical detection of AKI and probably contributes to the reduction in 1,25(OH)_2_D observed by Cameron et al. [1]. Interestingly, 1,25(OH)_2_D appears to increase briefly during or after AKI recovery. It would certainly be interesting and enlightening to evaluate the longitudinal behavior of FGF23.

Another aspect raised by the authors is the supplementation of active vitamin D in AKI. In our previously cited study [4], we suggested that FGF23 might be associated with endothelial damage and subsequent AKI development. Understanding the complex interactions between serum calcium, phosphorus, parathyroid hormone, FGF-23, and vitamin D metabolism is crucial for proposing potential preventative or therapeutic interventions focused on this intricate axis. In fact, this area warrants further longitudinal studies with serial measurements of all components involved in vitamin D metabolism.

## Data Availability

No datasets were generated or analysed during the current study.
